# Challenges in interpreting allergen microarrays in relation to clinical symptoms: A machine learning approach

**DOI:** 10.1111/pai.12139

**Published:** 2013-10-16

**Authors:** Mattia C F Prosperi, Danielle Belgrave, Iain Buchan, Angela Simpson, Adnan Custovic

**Affiliations:** 1 Centre for Health Informatics, Institute of Population Health, University of ManchesterManchester, UK; 2 Centre for Respiratory Medicine and Allergy, Institute of Inflammation and Repair, University of ManchesterManchester, UK

**Keywords:** component-resolved diagnostics, asthma, wheeze, rhinitis, airway hyper-reactivity, methacholine, IgE, children, machine learning, feature selection, logistic regression, random forests, Bayesian networks

## Abstract

**Background:**

Identifying different patterns of allergens and understanding their predictive ability in relation to asthma and other allergic diseases is crucial for the design of personalized diagnostic tools.

**Methods:**

Allergen-IgE screening using ImmunoCAP ISAC® assay was performed at age 11 yrs in children participating a population-based birth cohort. Logistic regression (LR) and nonlinear statistical learning models, including random forests (RF) and Bayesian networks (BN), coupled with feature selection approaches, were used to identify patterns of allergen responses associated with asthma, rhino-conjunctivitis, wheeze, eczema and airway hyper-reactivity (AHR, positive methacholine challenge). Sensitivity/specificity and area under the receiver operating characteristic (AUROC) were used to assess model performance *via* repeated validation.

**Results:**

Serum sample for IgE measurement was obtained from 461 of 822 (56.1%) participants. Two hundred and thirty-eight of 461 (51.6%) children had at least one of 112 allergen components IgE > 0 ISU. The binary threshold >0.3 ISU performed less well than using continuous IgE values, discretizing data or using other data transformations, but not significantly (p = 0.1). With the exception of eczema (AUROC∼0.5), LR, RF and BN achieved comparable AUROC, ranging from 0.76 to 0.82. Dust mite, pollens and pet allergens were highly associated with asthma, whilst pollens and dust mite with rhino-conjunctivitis. Egg/bovine allergens were associated with eczema.

**Conclusions:**

After validation, LR, RF and BN demonstrated reasonable discrimination ability for asthma, rhino-conjunctivitis, wheeze and AHR, but not for eczema. However, further improvements in threshold ascertainment and/or value transformation for different components, and better interpretation algorithms are needed to fully capitalize on the potential of the technology.

Detection of allergen-specific IgE antibodies (sIgE) is associated with an increased risk of wheeze/asthma, and among asthmatic patients with more severe disease and diminished lung function 1–4. The level of sIgE to common inhalant allergens offers more valuable information than a simple detection of ‘positive sIgE’ 4,5. Different allergen sources (both indoor and outdoor) have been independently associated with asthma and asthma-related symptoms 6–9. However, it remains unclear how allergen sensitizations *in toto* contribute towards clinical manifestations of different atopic diseases (e.g., asthma vs. rhino-conjunctivitis vs. eczema).

The increasing availability of allergen components (purified from natural source or produced as recombinant proteins) marks the shift in allergy diagnosis that may lead to a transition towards component-resolved diagnostics 10. For example, the multiplex chip-based assay ImmunoCAP ISAC® has been validated in terms of performance and reproducibility 11, providing an opportunity to identify both allergen patterns and their interactions in relation to different clinical outcomes. Using ImmunoCAP ISAC® assay, the sIgE antibody profiles associated with asthma, exhaled nitric oxide and airway hyper-reactivity (AHR) have been investigated, with multiple sensitizations to several allergen groups increasing the risk of asthma 12.

We hypothesized that different sIgE patterns are predictive of different diseases commonly associated with atopy; also, different interpretation algorithms, component threshold ascertainment and/or value transformation may modify the association between such patterns and clinical symptoms. To address these hypotheses, we investigated the ability and the interpretability of different linear and nonlinear statistical learning models in classifying contemporaneous asthma, wheezing, AHR, rhino-conjunctivitis and eczema. Models were fit on sIgE levels measured by the ISAC® among participants in a population-based birth cohort at age 11 yrs. We used machine learning methods such as decision trees (DTs) (that divide study population into nested subgroups, based on allergen thresholds and combinations, each with a specific probability of manifesting clinical symptoms) and Bayesian networks (BN) (graphs that represent causal dependencies of variables), coupled with logistic regression (LR), to fully exploit the large amount of information provided by the microarray, and to associate it to clinical symptoms.

## Methods

### Study population and data sources

Manchester Asthma and Allergy Study is a population-based birth cohort described in detail elsewhere 13. The study was approved by a local ethics committee; informed consent was obtained from all parents. All data in this manuscript were ascertained at age 11 yrs. We administered validated questionnaires to collect information on parentally reported symptoms and physician-diagnosed illnesses. We measured AHR using methacholine challenge 14.

### Definition of outcomes

#### Current asthma

Positive answer to all three of the following questions: (i) ‘Has your child wheezed within the past 12 months?’, (ii) ‘Has your child received asthma medication within the past 12 months?’ and (iii) ‘Has your child ever been diagnosed with asthma?’.

#### Current wheeze

Positive answer to the question ‘Has your child had wheezing or whistling in the chest in the last 12 months?’.

#### Current eczema

Positive answer to the question ‘Has your child had eczema in the last 12 months?’.

#### Current rhino-conjunctivitis

Positive answer to the question ‘In the past 12 months, has your child ever had a problem with sneezing, or a runny nose, or a blocked nose when he/she did not have a cold or the flu which was accompanied by itchy-watery eyes?’.

#### Airway hyper-reactivity

Provocative concentration of methacholine causing a 20% decline in FEV_1_ < 16 mg/ml.

All outcomes were encoded as binary variables. Other variables considered in the descriptive statistics were FEV_1_, FVC, FEV_1_/FVC ratio, eNO and methacholine dose-response ratio.

### Detection of IgE antibodies

The presence of sIgE to 112 allergen components was assessed by the ImmunoCAP ISAC® (ThermoFisher Scientific, Uppsala, Sweden).

#### Transformation of sIgE values

In the analyses described below, we expressed sIgE values as follows: (i) binarized using the threshold of 0.3 ISU; (ii) discretized into four categories using the manufacturer's semiquantitative scale (<0.3 ISU, undetectable or very low; ≥0.3 and <1 ISU, low; ≥1 and <15 ISU, moderate to high; ≥15 ISU, very high); (iii) discretized using an automated supervised discretization approach 15; (iv) continuous raw values; (v) square-root or hyperbolic-arcsine transformation 16; (vi) using other normalization methods such as quantile normalization 17.

### Statistical learning

For a detailed explanation of the methods, please see the Supporting Information. Briefly, we analysed the discriminative ability of sIgE patterns in relation to clinical outcomes by fitting a series of machine learning models. All analyses were adjusted for gender. Statistical models were run on the subset of patients with at least one sIgE > 0 ISU.

#### Logistic regression

We fitted main-effects LR using: (i) the sum of and the number of positive sIgE values; and (ii) sIgE to all allergen components (using different transformations). For the latter, due to the high number of variables, LR was subject to feature selection *via* LogitBoost 18.

#### Decision tree and random forest models

These models were fitted to investigate possible nonlinear/interaction effects 19,20. DTs are machine learning methods that divide population into nested subgroups according to values of the covariates, usually those that have the highest discriminatory power with respect to the outcome. For example, our study population could be divided into two using Fel d 1 IgE below or above 0.3 ISU. Other tree-branching rules can then be inferred on the two subpopulations, and so on recursively until a stopping criterion is met (e.g., a minimum number of subjects per subgroup). This progressive data partition can be represented in the form of a tree (Fig. S1). DTs are easy to interpret, but sometimes have poor predictive power. RFs are an ensemble of several different DTs, fitted with resampling/randomization, with the aim to improve prediction performance by combining many decision pathways (e.g., averaging across many DT predictions). DT and RF have dedicated methods for measuring variable importance which can capture complex interactions, without the need of explicitly defining them.

#### Bayesian networks

Bayesian networks are graphs in which each node is a covariate, and a link between two nodes represent a dependency. If no link is present between two nodes, they are conditionally independent. For a comprehensive introduction to BN modelling for biomedicine, please see Millán et al. 21. The naïve Bayes (NB), a simpler model which assumes conditional independence among variables, was also fit as a control to BN.

#### Model performance

Goodness-of-fit functions for assessing prediction performance of models included as follows: accuracy (% correct), area under the receiver operating characteristic (AUROC), sensitivity and specificity. The ability to generalize on unseen data was assessed through repeated validation, executing for 50 times a randomized training/test procedure (80%/20%) and comparing differences between models with a paired-corrected *t*-test. We also assessed power of the sample in relation to covariate size.

## Results

### Participants

We reviewed 822 children. Sample for IgE measurement was obtained for 461 (56.1%); there was no difference in gender, family history of allergic diseases, position in sibship, asthma, sensitization (skin tests) or parental atopy between those with and without IgE (data available on request). A total of 238 of 461 (51.6%) children tested positive (sIgE > 0.3 ISU) to at least one allergen component. Characteristics of study participants are shown in Table1.

**Table 1:** Characteristics of the study population at age 11 (N = 426)

At least one IgE > 0	Median (IQR) or N			Missing
	Male 154	Female 84	Total 238	N/A
N_1_ = 238 (51.6%)				
Number of specific positive IgE (>0.3 ISU)	7.5 (3.0–14.5)	7.0 (2.0–12.0)	7.0 (3.0–13.0)	N/A
Sum of all IgE	34.0 (8.4–130.2)	38.9 (1.9–132.8)	36.4 (4.9–131.4)	N/A
Asthma	33	15	48	4
Eczema	36	22	58	5
Mean eNO	14.6 (8.8–33.8)	19.9 (9.8–41.7)	17.6 (9.4–37.8)	62
FVC	2.7 (2.4–3.0)	2.5 (2.3–2.9)	2.6 (2.4–3.0)	3
% predicted FEV_1_	99.2 (91.0–107.4)	98.5 (92.2–103.7)	99.0 (91.6–106.0)	3
Current wheeze	48	25	73	3
AHR	62	31	93	59
Rhino-conjunctivitis	63	28	91	3
Methacholine dose-response ratio	3.20 (1.01–5.47)	3.48 (0.95–6.11)	0.98 (3.28–5.84)	57
FEV/FVC ratio	0.86 (0.81–0.90)	0.89 (0.84–0.92)	0.86 (0.82–0.91)	4

All IgE = 0	Male 101	Female 122	Total 223	N/A
N_2_ = 223 (48.4%)				
Asthma	5	5	10	9
Eczema	15	14	29	7
Mean eNO	8.3 (6.9–10.7)	8.1 (6.5–10.4)	8.2 (6.6–10.6)	46
FVC	2.7 (2.5–3.1)	2.6 (2.3–2.9)	2.7 (2.4–3.0)	6
% Predicted FEV_1_	98.3 (93.0–106.1)	99.0 (91.8–106.6)	98.6 (92.5–106.5)	5
Current wheeze	8	10	18	2
Airway hyper-reactivity	23	18	41	50
Rhino-conjunctivitis	7	9	16	11
Methacholine dose-response ratio	4.69 (2.88–6.48)	5.13 (3.81–6.81)	4.99 (3.54–6.57)	53
FEV/FVC ratio	0.86 (0.82–0.90)	0.89 (0.85–0.93)	0.88 (0.83–0.92)	13

### Distribution and transformation of sIgE values

The distribution of sIgE values was highly skewed; Fig. S2 shows the histograms upon several input transformations. In a preliminary test on model performance (AUROC in relation to asthma, using LR and RF), we found that the binary discretization of sIgE values using the 0.3 ISU threshold was performing less well compared with a continuous scale or a multiple categorization. The manufacturer's semiquantitative scale performed better than the binary threshold, although not significantly. The automated supervised discretization method yielded the best results. Fig. S3 shows detailed box plots of AUROC performance across all transformation methods.

### Discriminative ability of sIgE patterns in relation to clinical outcomes

#### Robustness of model performance

Average AUROC > 0.5 was achieved for asthma, wheeze, rhino-conjunctivitis and AHR in all statistical learning models; in contrast, this was not achieved for eczema (AUROC∼0.5). However, although AUROC for eczema for all models was poor, in the univariate analysis, sIgE to egg ovomucoid, ovalbumin and ovotransferrin were significantly associated with eczema (Gal d 1, p = 0.02; Gal d 2; p = 0.02; Gal d 3, p = 0.02). We also observed a strong trend for bovine allergens Bos d 4-5-6 (p = 0.06, p = 0.06, p = 0.04, respectively).

Overall, all models showed reasonable AUROC (0.76–0.82 for RF, 0.63–0.79 for LR, 0.56–0.77 for BN, 0.64–0.76 for NB) and sensitivity (0.69–0.97 for RF, 0.54–0.95 for LR, 0.58–0.96 for BN, 0.62–0.92 for NB), but poor specificity (0.34–0.70 for RF, 0.40–0.69 for LR, 0.32–0.54 for BN, 0.38–0.57 for NB), except for the positive AHR (higher specificity, decreased AUROC/sensitivity).

**Figure 1:**
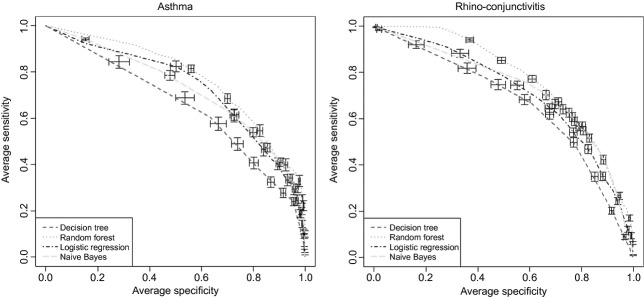
Performance of statistical learning models in classifying the asthma and rhino-conjunctivitis outcomes, using the full feature set (112 IgE + gender) by means of area under the receiver operating characteristic, across 50 independent validation (80%/20%) runs. Results are out-of-sample predictions (i.e., on unseen data). Bars represent standard errors.

Random forest outperformed other approaches in terms of AUROC in all but one outcome (rhino-conjunctivitis). LR (model ii) ranked always as the best in terms of specificity. The number of variables selected by LogitBoost yielded a median (IQR) of 9 7–18 covariates per model across all validation runs. In most cases, the hypothesis that there was no difference in the mean performance among the RF, LR, NB and BN could not be rejected at the 0.05 level. Performance of DT was consistently inferior to that of RF (p < 0.05), and the same held for LR model i (encoding the number of positive IgE + sum of all IgE), except for rhino-conjunctivitis. Table2 summarizes prediction performance obtained by the repeated validation; AUROC plots are shown in Fig.1. For this experiment, we used the square-root transformed sIgE, but similar results were obtained for the other transformation methods.

**Table 2:** Performance of statistical learning models by means of 50 independent validation runs, stratified by different outcomes

Outcome	Method	Feature set	Feature/topology selection	AUROC (s.d.)	Sensitivity (s.d.)	Specificity (s.d.)
Asthma	Majority class	N/A	N/A	0.50 (0.00)*	**1.00 (0.00)**	0.00 (0.00)*
	LR	Number of positive IgE + sum of all IgE	N/A	0.71 (0.10)*	0.96 (0.03)	0.20 (0.12)*
	LR	112 IgE + gender	Cross-validated LogitBoost	0.79 (0.08)	0.95 (0.03)*	**0.40 (0.15)**
	DT	112 IgE + gender	Embedded (information gain, pruning)	0.59 (0.10)*	0.96 (0.06)	0.14 (0.16)
	RF	112 IgE + gender	Embedded (Gini index, random subset)	**0.82 (0.06)**	0.97 (0.04)	0.34 (0.13)
	NB	112 IgE + gender	Cross-validated wrapper (best-first search, K2)	0.76 (0.08)	0.91 (0.05)*	0.38 (0.15)
	BN	112 IgE + gender	Cross-validated wrapper (best-first search, K2)	0.77 (0.07)	0.96 (0.03)*	0.32 (0.15)
Wheeze	Majority class	N/A	N/A	0.50 (0.00)*	**1.00 (0.00)**	0.00 (0.00)*
	LR	Number of positive IgE + sum of all IgE	N/A	0.67 (0.06)*	0.93 (0.04)*	0.13 (0.08)*
	LR	112 IgE + gender	Cross-validated LogitBoost	0.72 (0.07)	0.94 (0.05)	**0.37 (0.12)**
	DT	112 IgE + gender	Embedded (information gain, pruning)	0.61 (0.08)*	0.90 (0.09)	0.29 (0.20)
	RF	112 IgE + gender	Embedded (Gini index, random subset)	**0.78 (0.06)**	0.91 (0.05)*	0.45 (0.12)
	NB	112 IgE + gender	Cross-validated wrapper (best-first search, K2)	0.69 (0.06)*	0.92 (0.06)*	0.30 (0.12)
	BN	112 IgE + gender	Cross-validated wrapper (best-first search, K2)	0.65 (0.07)*	0.93 (0.10)	0.29 (0.12)
Rhino-conjunctivitis	Majority class	N/A	N/A	0.50 (0.00)*	**1.00 (0.00)**	0.00 (0.00)*
	LR	Number of positive IgE + sum of all IgE	N/A	**0.80 (0.07)**	0.84 (0.07)*	0.44 (0.11)*
	LR	112 IgE + gender	Cross-validated LogitBoost	0.73 (0.07)*	0.79 (0.09)*	**0.53 (0.13)**
	DT	112 IgE + gender	Embedded (information gain, pruning)	0.66 (0.06)*	0.81 (0.11)*	0.47 (0.17)
	RF	112 IgE + gender	Embedded (Gini index, random subset)	0.78 (0.07)	0.80 (0.08)*	0.57 (0.11)
	NB	112 IgE + gender	Cross-validated wrapper (best-first search, K2)	0.75 (0.07)	0.88 (0.06)*	0.41 (0.12)*
	BN	112 IgE + gender	Cross-validated wrapper (best-first search, K2)	0.73 (0.07)*	0.82 (0.08)*	0.50 (0.12)
AHR	Majority class	N/A	N/A	0.50 (0.00)*	**1.00 (0.00)**	0.00 (0.00)*
	LR	Number of positive IgE + sum of all IgE	N/A	0.57 (0.11)*	0.70 (0.19)*	0.39 (0.20)*
	LR	112 IgE + gender	Cross-validated LogitBoost	0.64 (0.05)*	0.55 (0.18)*	0.70 (0.23)
	DT	112 IgE + gender	Embedded (information gain, pruning)	0.64 (0.10)*	0.55 (0.18)*	0.70 (0.21)
	RF	112 IgE + gender	Embedded (Gini index, random subset)	**0.76 (0.07)**	0.69 (0.11)*	**0.70 (0.10)**
	NB	112 IgE + gender	Cross-validated wrapper (best-first search, K2)	0.64 (0.10)*	0.62 (0.20)*	0.57 (0.25)
	BN	112 IgE + gender	Cross-validated wrapper (best-first search, K2)	0.56 (0.06)*	0.58 (0.21)*	0.54 (0.29)

AHR, airway hyper-reactivity; AUROC, area under the receiver operating characteristic; BN, Bayesian networks; DT, Decision tree; LR, Logistic regression; NB, naïve Bayes; RF, random forests.

* The hypothesis of difference in means comparing against the best model (in bold) could not be rejected at p = 0.05.

#### Variable importance in relation to clinical outcomes and their dependencies

**Figure 2:**
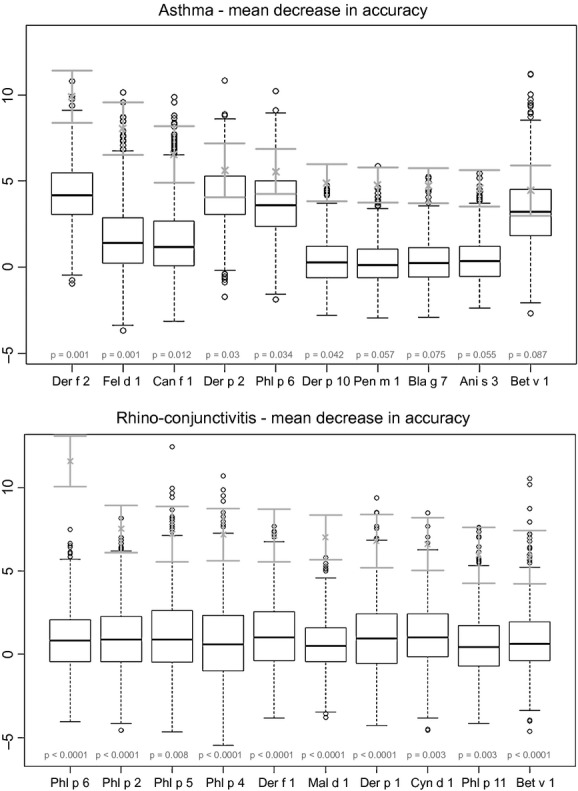
Feature importance plots for the asthma outcome (upper panel) and for the rhino-conjunctivitis outcome (lower panel) measured as mean decrease in accuracy from fitting a random forest and performing an outcome permutation test (1000 runs). Green intervals represent rescaled average (±standard deviation) decrease in accuracy, whilst box plots represent the null distribution (randomized outcomes); p-values are highlighted in red. Only the first 10 variables shown.

Fig.2 shows RF feature importance plots with respect to asthma and rhino-conjunctivitis across 1000 permutation runs. The importance is expressed as rescaled decrease in accuracy when randomizing a variable of interest. For asthma, there was a broader set of top-scoring allergens belonging to different sources including dust mite, cat, dog and pollens, whilst the top-scoring allergens for rhino-conjunctivitis were all pollens followed by dust mite. The order of variables may change when randomizing permutations 22; indeed, variable ranks and associated p-values for our data set were subject to a consistent degree of variation.

**Figure 3:**
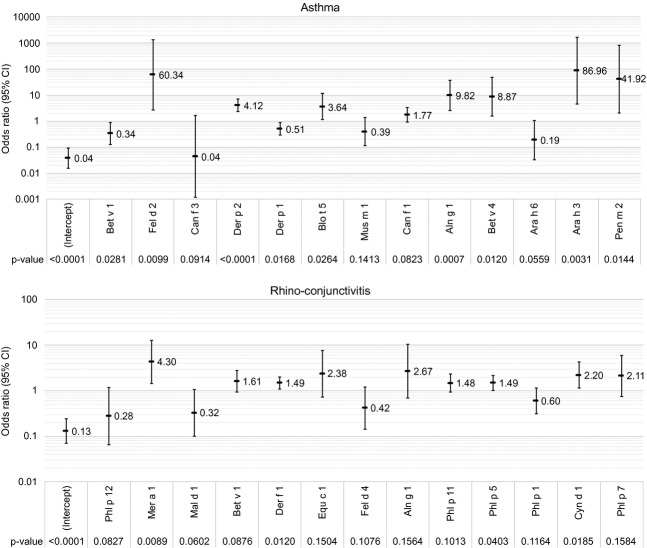
Multivariable logistic regression for asthma and rhino-conjunctivitis outcomes (upper and lower panel, respectively), showing mutually adjusted odds ratios and associated p-values from the LogitBoost algorithm (run on the whole data set). Only variables significant in the univariate analysis were included (p < 0.05).

There was partial consistency between the variables selected by the stepwise heuristic for NB/BN with the top-scoring variables output by the RF. Similarly, runs of stepwise LR with different starting points led to different final sets, probably due to a number of correlated variables. Fig.3 shows the mutually adjusted odds ratio from LogitBoost LR, including only variables significant (p < 0.05) in univariate analysis. Supplementary results give a more thorough explanation of the relevant variables and their association into ‘equivalency’ groups (Figs S4–S6).

**Figure 4:**
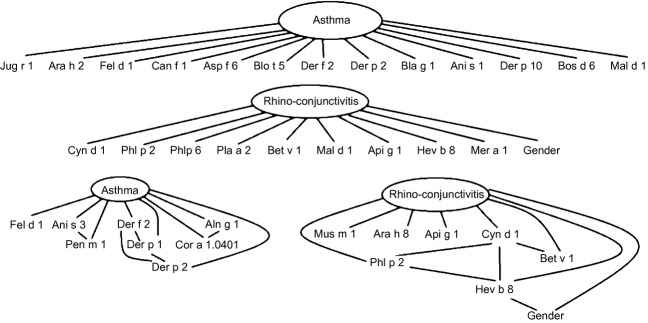
Bayesian networks (BN) for the classification of asthma and rhino-conjunctivitis. Upper panel shows the naïve Bayes (NB) models (hypothesizing variable independence, it can be abstracted to a main-effect logistic model, that is, a linear score where each variable has a weight), and lower panel shows the BN that allow for more complex (direct and indirect) conditional dependencies. Given the non-superiority of the more complex BN model as compared to the NB on the current data set, one could choose the NB hypothesis and further evaluate different variable sets, as in main-effects logistic regression.

Fig.4 depicts two optimized NB and BN structures for asthma and rhino-conjunctivitis. The networks shown here are representative of a single run; therefore, we cannot exclude the possibility that there are other topologies and variable sets with comparable performance.

## Discussion

### Key findings

We investigated the ability of linear and nonlinear statistical learning models fit on ISAC® assay data, to identify asthma, wheezing, AHR, rhino-conjunctivitis and eczema. In general, all modelling techniques (excluding DRs) performed comparably. With the exception of eczema, all outcomes could be predicted with an AUROC > 0.5. Random forests (RF) outperformed other approaches in terms of AUROC, whilst LR ranked as the best in terms of specificity. We could not clarify if a main-effect model or a model that hypothesizes conditional independence among variables (LR/NB) was performing as well as a model which accounts for interactions or conditional dependencies (RF/BN). Based on these results, one could argue that a simple linear score (LR) with fewer allergen components (from 7 to 18) may be as effective as a more complex model. However, we cannot rule out the possibility that interactions among allergens may have a potentially important role.

Our data suggest that sIgE discretization and transformation policy may increase the model performance compared with the single dichotomous threshold at 0.3 ISU. The number of positive sIgEs and the sum of all sIgE levels were poorer predictors of asthma, wheeze and AHR compared with the information on all components, supporting the evaluation of all specific components rather than an overall qualitative assessment.

### Limitations and interpretation

From a methodological point of view, one limitation of this study lies within the procedures for feature/model selection. A challenge within BN learning is the simultaneous estimation of both node set and topology, limited here by the usage of two nested heuristic searches. Reliability of the associations in the networks was not assessed, nor the stability of selected feature sets. The selection of main effects and interactions might be also dependent on the discretization policy.

The advantage of coupling machine learning methods with classical statistical models is that more complex mechanistic hypotheses can be investigated and that large, sparse, heterogeneous data sets can be analysed. Furthermore, by comparing performance of nonlinear and linear methods, one can ascertain whether the unexplained variability (e.g., poor prediction of the outcome) can be reduced by measuring other potentially important variables, permitting formulation of new hypotheses.

Our data suggest that a careful and informed sIgE discretization/transformation may increase the performance, although the differences in AUROC of various approaches could not be always confirmed at the formal 0.05 significance level. The semiquantitative coding suggested by the manufacturer performed better than using the binary threshold at 0.3 ISU (albeit not significantly). In our data set, the automated supervised discretization method yielded the best results, supporting the notion that a range of population-specific (and perhaps age-specific) expected values should be established for different populations. Given the multimodal and skewed nature of sIgE distributions and relatively modest sample size, further analyses in larger data sets are warranted. We cannot exclude the possibility that different thresholds are applicable to different components.

The heuristic algorithms for attribute selection yielded compact sets of predictors (from hundreds to a dozen, confirmed when applying the automated supervised discretization approach), which simplifies the interpretation of the results. Not surprisingly, we confirmed previous findings 12 that mite, pollen and pet allergens are top-scoring predictors of asthma, whilst pollens (and mite) are for rhino-conjunctivitis. However, execution of the feature selection algorithms multiple times led to variations in the sets and scores. This may be in part due to highly correlated variables, but also other latent causes. Such variability does not permit identification of one unique model, and therefore, a unique pathway. Our results suggest that pre-clustering of allergens into relevant families may help stabilize the feature selection phases and may potentially be more useful than standard classifications by source (e.g., pollen or mite).

The prediction models for eczema had poor performance, suggesting either that disruption in skin barrier function may be more important in the pathogenesis of eczema than IgE-mediated mechanisms or that other allergens not available on the chip may be predictive of eczema (e.g., *Staphylococcus aureus* enterotoxins) 23.

## Conclusions

Component-resolved diagnostic tests may offer a more accurate assessment of allergic diseases. However, further improvements in threshold ascertainment and/or value transformations for different allergen components, well-thought-out interpretation algorithms and selection of components are needed to fully capitalize on the potential of the technology.

## Authors contributions

MCFP machine learning modelling, manuscript writing; DB data extraction, statistics, statistical review, manuscript review; AS study cohort management, experimental set up, manuscript review; AC principal investigator, study design, manuscript review; IB statistical review, manuscript review.

## Conflict of interest

The authors declare no conflict of interest in relation to this study.

## Funding

MAAS is supported by grants from J P Moulton Charitable Foundation, and MRC Grants G0601361 and MR/K002449/1. Study was partly supported by the University of Manchester's Health Research Center (HeRC) funded by the Medical Research Council (MRC) Grant MR/K006665/1. ImmunoCAP ISAC® assay was performed by the ThermoFisher Scientific, Uppsala, Sweden, as an in-kind contribution; ThermoFisher Scientific had no role in study design, data collection, analysis, interpretation, writing of the report or the decision to submit the study for publication.

## Supporting Information

Figure S1 Example of a decision tree, classifying an outcome of interest (1/2) based on the values of variables *x*, *y* and *z*. **Figure S2.** Distribution of IgE values from the ISAC chip (ISU scale) by selecting all (left panels) and strictly positive (>0, right panel) values. **Figure S3.** Performance of RF and LR models by varying the input IgE transformation function, specifically (from left to right): binary 0.3 ISU threshold (blue colour), column-wise quartile discretisation, matrix-wise quartile discretisation, square-root transform, raw values, supervised discretisation, matrix-wise standardisation, quantile normalisation, and manufacturer's semi-quantitative scale. **Figure S4.** Correlation plots for allergen IgEs. Spearman rank-correlation was used, and the correlation matrix has been sorted to group highly-correlated allergen groups. **Figure S5.** Hierarchical clustering of allergen IgEs. Manhattan (L1 norm) distance was used, aggregating instances progressively with the complete linkage method. **Figure S6.** Thresholded adjacency graph of allergen IgE, based on Spearman's rank-correlation.
