# Supplementary material for: Challenges in interpreting allergen microarrays in relation to clinical symptoms: A machine learning approach
Source: Pediatr Allergy Immunol. 2013 Oct 16;25(1):71–9. doi: 10.1111/pai.12139 (PMC4282342; doi:10.1111/pai.12139)
Supplement: Figure S1 — Example of a decision tree, classifying an outcome of interest (1/2) based on the values of variables x, y and z. Figure S2. Distribution of IgE values from the ISAC chip (ISU scale) by selecting all (left panels) and strictly positive (>0, right panel) values. Figure S3. Performance of RF and LR models by varying the input IgE transformation function, specifically (from left to right): binary 0.3 ISU threshold (blue colour), column-wise quartile discretisation, matrix-wise quartile discretisation, square-root transform, raw values, supervised discretisation, matrix-wise standardisation, quantile normalisation, and manufacturer's semi-quantitative scale. Figure S4. Correlation plots for allergen IgEs. Spearman rank-correlation was used, and the correlation matrix has been sorted to group highly-correlated allergen groups. Figure S5. Hierarchical clustering of allergen IgEs. Manhattan (L1 norm) distance was used, aggregating instances progressively with the complete linkage method. Figure S6. Thresholded adjacency graph of allergen IgE, based on Spearman's rank-correlation. [file pai0025-0071-sd1.docx]

**CHALLENGES IN INTERPRETING ALLERGEN MICROARRAYS IN RELATION TO CLINICAL SYMPTOMS: A MACHINE LEARNING APPROACH**

**SUPPLEMENTARY MATERIAL**

**METHODS**

***Study design:*** Unselected birth cohort study.

***Setting:*** Maternity catchment area of Wythenshawe and Stepping Hill Hospitals, a stable mixed urban-rural population.

***Screening & Recruitment:*** All pregnant women were screened for eligibility at antenatal visits (8^th^-10^th^ week of pregnancy). Of the 1,499 couples who met the inclusion criteria, 288 declined to take part in the study and 27 were lost to follow-up between recruitment and birth of a child. A total of 1,184 participants had at least some evaluable data.

***Clinical Follow-up:*** Children attended review clinic at age 11 years. A validated ISAAC questionnaire was interviewer-administered.

***Airway hyper-reactivity:*** Airway reactivity was assessed by methacholine challenge using a 5 step protocol performed according to ATS guidelines. Quadrupling doses of methacholine (0.0625–16.0 mg/mL) were delivered to subjects via a DeVilbiss 646 nebuliser (Sunrise Medical HHG, Somerset, PA) and a KoKo dosimeter (Pulmonary Data Services, Doylestown, PA) calibrated to deliver 0.009 mL per 0.6s actuation. The predicted FEV_1_ was calculated and if the measured value was <1.0 L or less than 60% predicted the test was not performed. FEV_1_ was measured 30 and 90 seconds after 5 inhalations of each dose of methacholine. The challenge was stopped when either a 20% fall in FEV_1_ was observed, or the maximum methacholine concentration had been administered.

***Statistical learning***

Logistic regression (LR)

We fitted main-effects logistic regression (LR) models using (i) either the sum of and the number of positive sIgE values, or (ii) sIgE to all allergen components. For the latter, due to the high number of variables involved, LR was subject to feature selection *via* cross-validated LogitBoost (1). We expressed sIgE values as:

1. discretised using the threshold of 0.3 ISU (binary)
2. discretised using the manufacturer’s semi-quantitative scale as: 0=undetectable or very low,<0.3 ISU; 1=low, ≥0.3 and <1 ISU; 2=moderate to high, ≥1 and <15 ISU; 3=very high, ≥15 ISU).
3. discretised using an automated supervised discretisation approach (2)
4. continuous raw values
5. their square-root/hyperbolic-arcsine transformation (3)
6. other microarray normalisation methods such as quantile normalisation (4)

Decision tree (DT) and random forest (RF) models

*Decision trees* are machine learning methods that divide the population into nested subgroups according to values of the covariates, usually those that have the highest discriminatory power with respect to the outcome of interest (e.g. according to a chi-square test). For instance, the original population of children in our study could be divided into two subgroups according to a Fel d 1 IgE value below or above 0.3 ISU. Then other tree-branching (or “split”) rules can be inferred on the two sub-populations, and so on recursively until a stopping criterion (called “pruning”) is met (e.g. a minimum number of subjects per subgroup). This progressive data partition can be represented in the form of a tree (Figure S1). Prediction rules are placed at the leaves of this tree (usually a constant value or a class category, but also a linear combination of input covariates). DTs are therefore easy to interpret, but sometimes have poor predictive power. In the current study, we based DT splits on information gain; pruning (removing sections of the tree that provide little power to classify instances) was used to reduce over-fitting.

*Random forests* are ensemble of several different DTs, fitted with variable parameters, with the aim to improve prediction performance by combining many decision pathways, e.g. averaging across all tree predictions. Specifically, a single DT within a RF is grown on a bootstrap sample of the original population, and splits are calculated in the same way except for the fact that only a random subset of variables is considered at each split, and no pruning is performed. In a RF, predictions are calculated by combining all single predictions as output by its internal DTs. Usually the average/median of all predictions or the majority vote is used. Here, RF was set up with 500 non-pruned random trees and √*P* features randomly selected at each split. Feature importance for RF was measured as rescaled mean decrease in accuracy (i.e. mean decrease divided by standard error) by considering RF fits randomising a variable of interest. Importance values were compared against a permutation of the outcome labels (1,000 times) in order to ascertain bias coming from different categorical scales or multimodal distributions (5), selecting variables with an average p-value≤0.05 according to the novel method of Altmann *et al.* (6). The advantage of using this method for variable importance evaluation instead of, for example, showing odds ratio from main-effects LR, resides in the fact that RF can capture complex variable interactions without the need of explicitly defining them. Therefore, the importance of a variable that has an effect only in combination with one other (or more) can be assessed.

Bayesian Networks (BN)

*Bayesian Networks* were fit here with the aim to describe in an interpretable way (i.e. via graphs) both the variables directly associated with the outcomes and the indirect dependencies across variables and outcomes. BN are directed acyclic graphs in which each node is a covariate and a directed link between two nodes represent a causal relation. If no link is present between two nodes, then they are conditionally independent. Therefore, a variable is uniquely determined by the values of its parents in the network. A “triangulation” or a “cascade” of links can be interpreted as the presence of confounding factors or indirect associations. A BN can be oriented towards an outcome node and inference can be made. With some restrictions (given by unobserved variables and network topology characteristics), a BN explains causal relationships, and even under relaxed hypotheses a BN is able to represent direct/indirect associations among variables. An intuitive and comprehensive introduction of BN modelling for biomedical sciences has been given by Millán et al. (7).

The *naïve Bayes* (NB), a simpler model which assumes conditional independence among variables, was also fit as a control to BN. NB is “conceptually” equivalent to a main-effect LR model. In fact, although NB and LR optimise different loss functions, under certain circumstances the two may converge to the same solution (8). Instead, a BN relaxes the independence assumption; each node variable is independent of its non-descendants in the graph given the state of its parents.

The topology optimisation of BN was achieved using the K2 heuristic algorithm (9), scoring topologies by means of the Bayesian Information Criterion (BIC), and setting the maximum numbers of parents to five. Feature selection for both BN and NB was done by 5-fold cross-validated wrapper method (10), subject to a greedy hill climbing search over the space of attribute subsets augmented with a backtracking facility (11).

Model performance

Goodness-of-fit functions for assessing prediction performance were:

1. area under the receiver operating characteristic (AUROC), which is equal to the probability that a classifier ranks a randomly chosen positive instance higher than a randomly chosen negative one;
2. sensitivity, i.e. the probability that a test result is positive when the condition is present (true positive rate);
3. specificity, i.e. the probability that a test result is negative when the condition is not present (true negative rate) (12).

Sensitivity and specificity were calculated using a fixed output probability threshold of 0.5. The robustness of model performance was analysed *via* repeated validation, and the goodness-of-fit distributions from test splits were compared using the Nadeau and Bengio’s t-test, which accounts for a modification of the degrees of freedom given the entity of sample overlap (13,14).

Sample size

Analytic formulation for determining the sample size for DT and RF is not available (15). For LR, we used the formula of Peduzzi *et al.* (16) where the sample size N=10*k/p (k being the number of covariates and p the smallest proportion of negative/positive cases in the outcome variable); therefore, with 238 cases, up to 5 covariates can be put in a LR model (simulations made at the 90% power reference).

Variable correlation and cluster analysis

In order to study the effect of correlated variables in the models, we employed unsupervised learning techniques, specifically: hierarchical clustering, rank-correlation analysis (17), and network analysis (18).

**RESULTS**

**Distribution of sIgE values**

The distribution of IgE values (after excluding columns with constant zeros) was highly skewed: by dividing it into zero and non-zero values (24,276 vs. 2,478), the latter appeared symmetrical if log-transformed, yet failing the Shapiro-Wilk test for normality, with a median (interquartile, IQR) range of 2.5 (0.65-10.5) ISU. Figure S2 shows the histograms of zero and non-zero values, upon several input transformations.

**Transformation of sIgE values**

By a preliminary test on variable encodings subject to model performance (AUROC with respect to asthma, using LR and RF), we found that the binary discretisation of IgE values using the 0.3 ISU threshold was performing less well than using a continuous scale or a multiple categorisation (e.g. automated supervised discretisation approach, square-root transform, normalisation of the raw IgE values, et cetera). The automated supervised discretisation method yielded the best results and categorised 17/112 variables with a non-constant indicator, excluding the others, thus this discretisation worked as a preliminary feature selection. However, the decrease in AUROC average of all input transformations as compared to that of the supervised discretisation approach could not be considered significant at the 0.05 level (p-value=0.1, comparing binary 0.3 threshold vs. supervised discretisation). The manufacturer’s semi-quantitative scale was better than the binary threshold as well, although not significantly.

**Statistical learning models for association with clinical outcomes**

Figure 4 in the manuscript depicts two optimised naïve Bayes and Bayesian network structures for asthma and rhino-conjunctivitis. The NB model hypotheses variable independence, and as such can be abstracted to a main-effect logistic model (i.e. a linear score where each variable has a weight). The BN allows more complex (direct and indirect) conditional dependencies. Given the non-superiority of the more complex BN model as compared to the NB on the current data set, one could choose the NB hypothesis and further evaluate different variable sets.

**Variable correlation and cluster analysis**

Since all the modelling techniques yielded non uniform feature sets over different validation runs, we sought to ascertain if this was partly due to highly-correlated variables. By applying rank-correlation analysis (Figure S4), hierarchical clustering (Figure S5) and network analysis (Figure S6), we identified several clusters of variables with a high-mutual association. The association was confirmed by using different metrics, including the Spearman’s rank-correlation and the Manhattan distance. The most stable groups were:

1. Cyn d 1, Phl p 1, Phl p 2, Phl p 4, Phl p 5, Phl p 6
2. Der f 1, Der f 2, Der p 1, Der p 2
3. Bet v 2, Hev b 8, Mer a 1, Phl p 12
4. Bet v 4, Phl p 7
5. Ani s 3, Bla g 7, Der p 10, Pen m 1
6. Ara h 1, Ara h 2, Ara h 6
7. Aln g 1, Bet v 1, Cor a 1.0101, Cor a 1.0401, Mal d 1, Pru p 1

These groups partly explain the variability in the feature sets selected by LR or BN/NB, and the large range of importance values yielded by the RF. For instance, referring to allergens of the group (g), Cor a 1.0401 was the variable with the highest association (p=0.00003) with the asthma outcome. Indeed, Cor a 1.0401 was not always selected by LogitBoost LR (only 20% of times across all validation runs), but alternatively Bet v1 and Aln g1 could be picked up. Likewise, the RF ranked Bet v 1 among the top-scoring variables, whilst Mal d 1 was selected by the NB model. In fact, the BN detected an association of both Aln g 1 and Cor a 1.0401 with the outcome, and also an association between the two allergens.

**Figure S1.** Example of a decision tree, classifying an outcome of interest (1/2) based on the values of variables x, y and z. At each tree node the population is divide into subgroups according to a test on the variable split. The leaf nodes represent classification rules calculated from the root to the tip of the tree. For instance the rightmost pathway represent the rule “IF (condition c for variable x is met) AND IF (condition g for variable z is met) THEN (probability of outcome 1 is 97%)”. Therefore the tree is able also to code variable interactions.


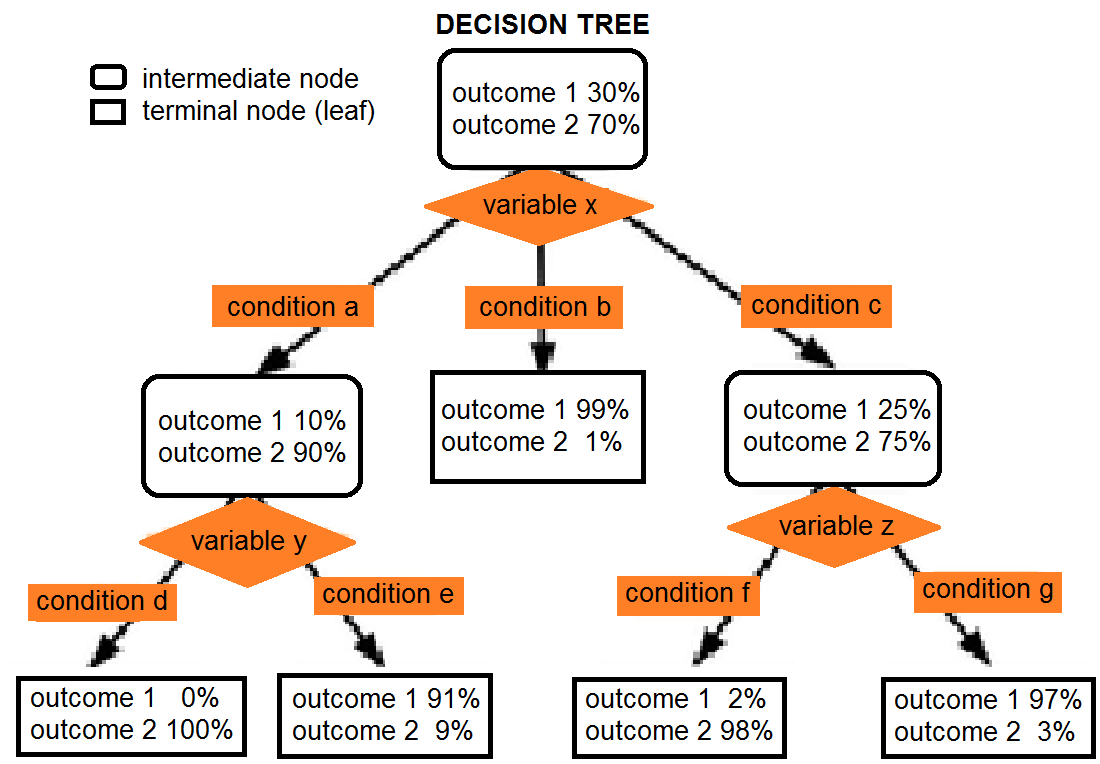


**Figure S2.** Distribution of IgE values from the ISAC chip (ISU scale) by selecting all (left panels) and strictly positive (>0, right panel) values. Different non-linear transformations are shown.


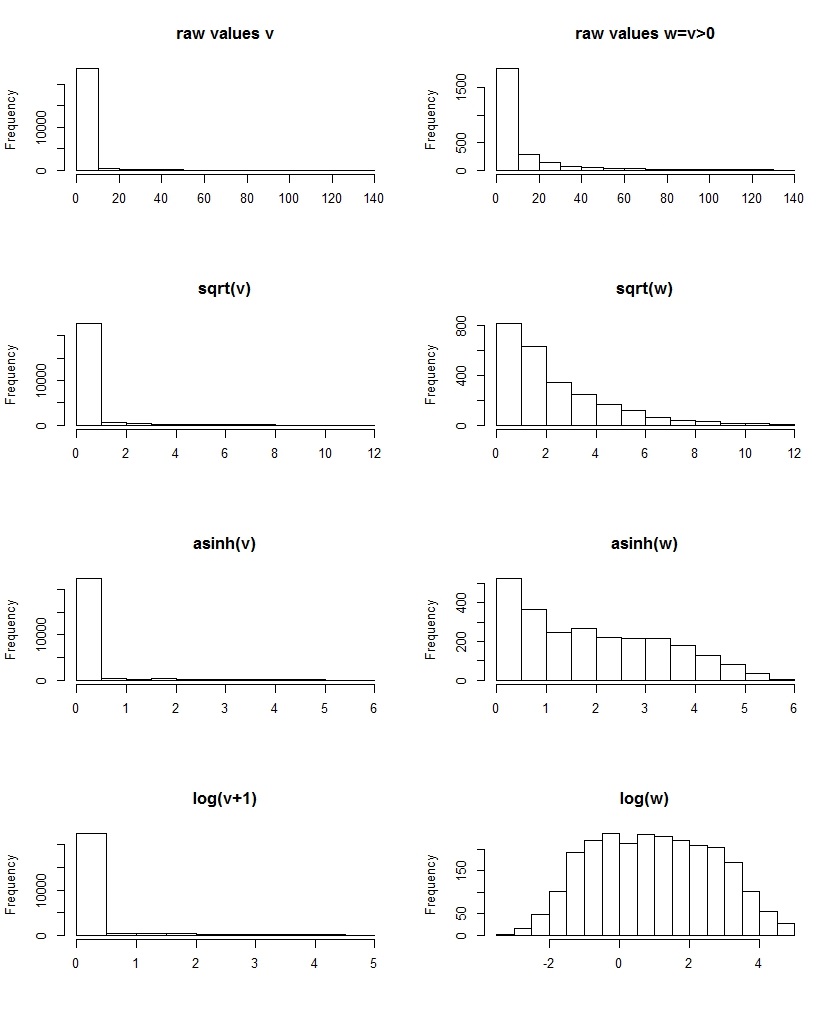


**Figure S3.** Performance of RF and LR models by varying the input IgE transformation function, specifically (from left to right): binary 0.3 ISU threshold (blue colour), column-wise quartile discretisation, matrix-wise quartile discretisation, square-root transform, raw values, supervised discretisation, matrix-wise standardisation, quantile normalisation, and manufacturer’s semi-quantitative scale. Boxplots represent out-of-bag AUROCs averaged across 50 bootstrap runs, and p-values are calculated with respect to the RF trained using the supervised discretisation (the best model in terms of AUROC). Green boxplots are from RF and LR trained on the supervised discretisation, whilst blue boxplots represent AUROCs from the binary discretisation at 0.3 ISU (p-value=0.1). Red boxplots refer to the manufacturer’s semi-quantitative scale.


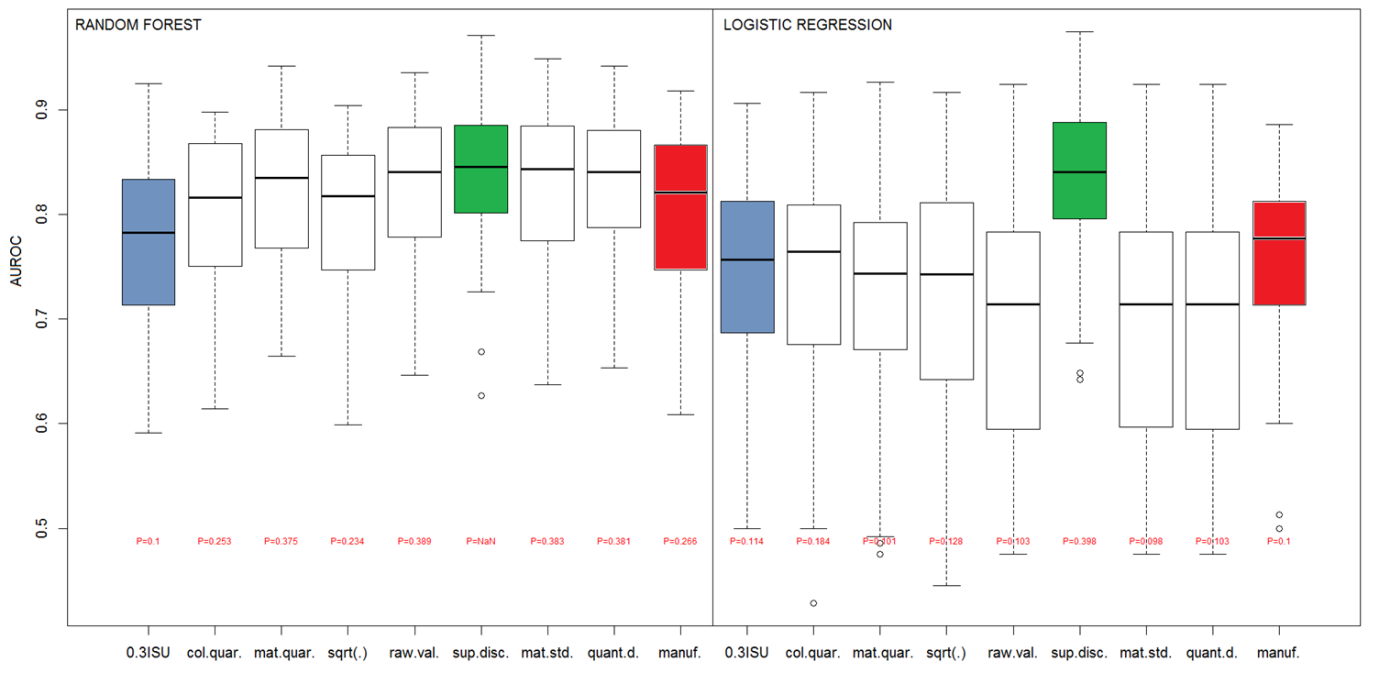


**Figure S4.** Correlation plots for allergen IgEs. Spearman rank-correlation was used, and the correlation matrix has been sorted to group highly-correlated allergen groups. Deep blue represents high-level correlation, deep red is the opposite, whilst white means no correlation.


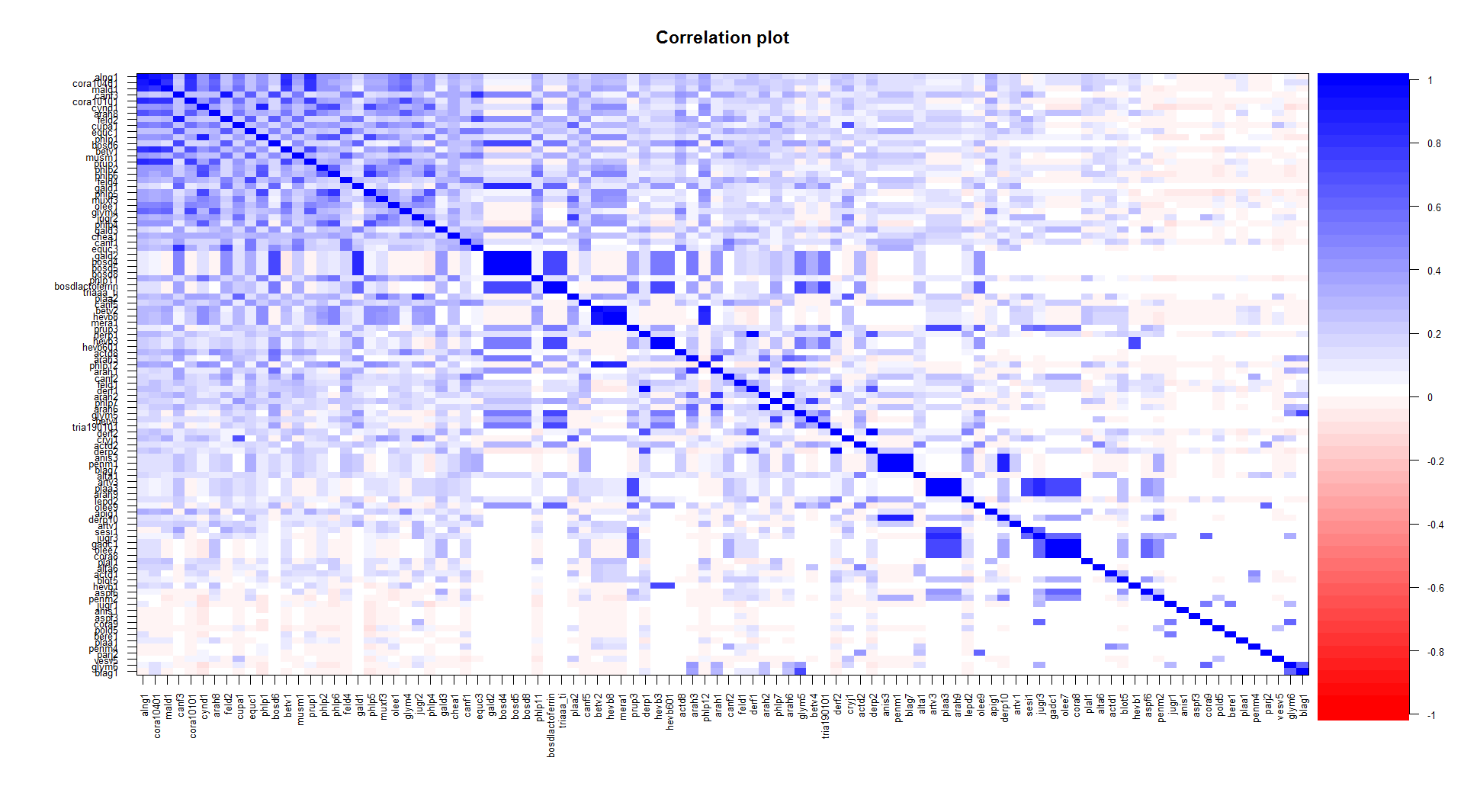


**Figure S5.** Hierarchical clustering of allergen IgEs. Manhattan (L1 norm) distance was used, aggregating instances progressively with the complete linkage method. Bootstrap analysis was performed (500 resampled sets), and node reliability is indicated in % as red/green p-values/bootstrap support.


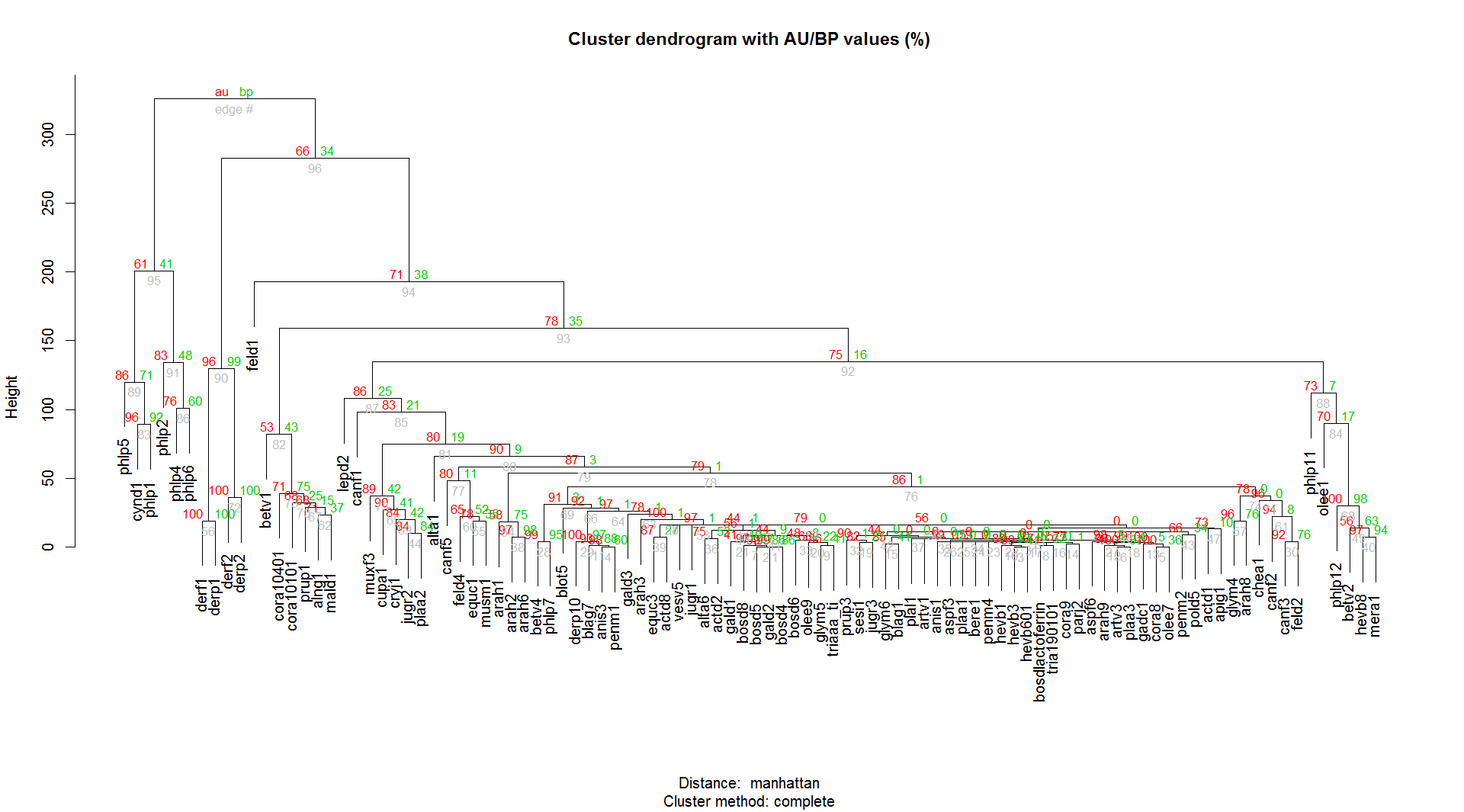


**Figure S6.** Thresholded adjacency graph of allergen IgE, based on Spearman’s rank-correlation. Variables with a correlation >95^th^ percentile of the whole set of correlations across all data are connected. Singletons represent variables that have no relevant correlation with the others.


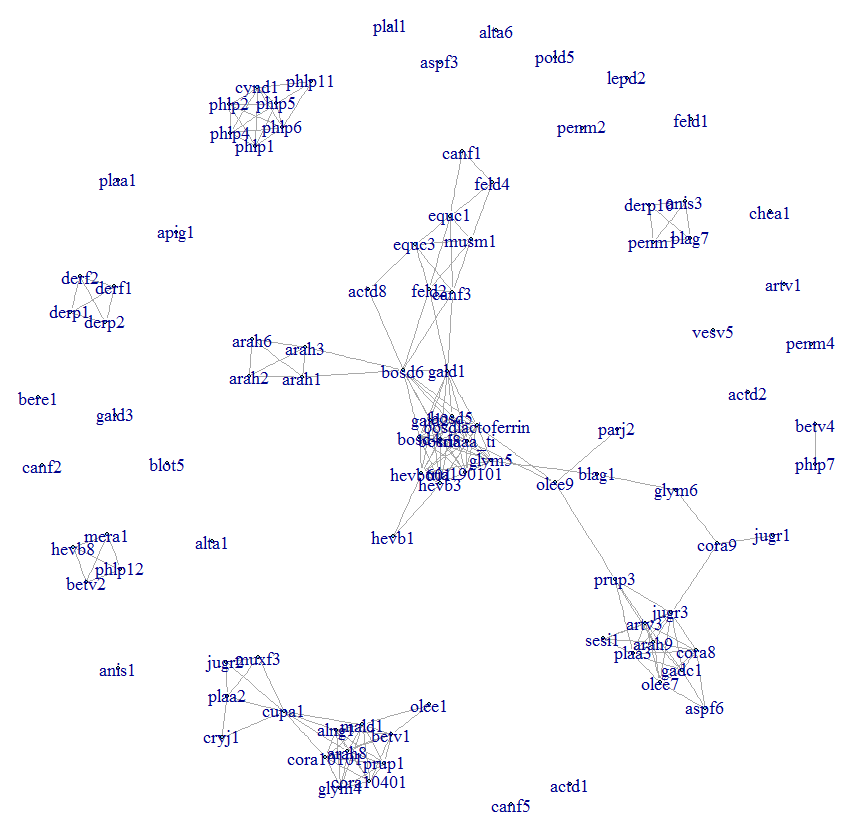


**REFERENCES**

1. Friedman J, Hastie T, Tibshirani R. Additive logistic regression: a statistical view of boosting (With discussion and a rejoinder by the authors). *The Annals of Statistics* 2000;**28**:337–407.

2. Fayyad UM, Irani KB. Multi-Interval Discretization of Continuous-Valued Attributes for Classification Learning. In: Bajcsy R, ed. *Proceedings of the International Joint Conference on Uncertainty in AI*. Morgan Kaufmann 1993. 1022–7.

3. Huber W, Von Heydebreck A, Sültmann H, Poustka A, Vingron M. Variance stabilization applied to microarray data calibration and to the quantification of differential expression. *Bioinformatics* 2002;**18 Suppl 1**:S96–S104.

4. Bolstad BM, Irizarry RA, Astrand M, Speed TP. A comparison of normalization methods for high density oligonucleotide array data based on variance and bias. *Bioinformatics* 2003;**19**:185–93.

5. Strobl C, Boulesteix AL, Zeileis A, Hothorn T. Strobl, Boulesteix, Zeileis, Hothorn: Bias in Random Forest Variable Importance Measures: Illustrations, Sources and a Solution. *Online* 2006;**490**:1–6.

6. Altmann A, Toloşi L, Sander O, Lengauer T. Permutation importance: a corrected feature importance measure. *Bioinformatics* 2010;**26**:1340–7.

7. Millán E, Loboda T, Pérez-de-la-Cruz JL. Bayesian networks for student model engineering. *Computers & Education* 2010;**55**:1663–83.

8. Ng AY, Jordan MI. On discriminative vs. generative classifiers: A comparison of logistic regression and naive bayes. *Advances in neural information processing systems* 2002;**2**:841–8.

9. Cooper GF, Herskovits E. A Bayesian method for the induction of probabilistic networks from data. *Machine Learning* 1992;**9**:309–47.

10. Kohavi R, John GH. Wrappers for feature subset selection. *Artificial Intelligence* 1997;**97**:273–324.

11. Russell SJ, Norvig P. Artificial Intelligence, A Modern Approach - 2nd Edition.pdf. Artificial Intelligence. 2003;**82**:874.

12. Hastie T, Tibshirani R, Friedman J. The Elements of Statistical Learning. *Elements* 2009;**1**:337–87.

13. Bengio Y, Grandvalet Y. Bias in Estimating the Variance of K-Fold Cross-Validation. In: Duchesne P, Remillard B, eds. *Statistical Modeling and Analysis for Complex Data Problem*. Springer 2005. 75–95.

14. Nadeau C, Bengio Y. Inference for the Generalization Error. *Machine Learning* 2003;**52**:239–81.

15. Sug HSH. A Structural Sampling Technique for Better Decision Trees. Ieee 2009.

16. Peduzzi P, Concato J, Kemper E, Holford TR, Feinstein AR. A simulation study of the number of events per variable in logistic regression analysis. *Journal of Clinical Epidemiology* 1996;**49**:1373–9.

17. Hahne F, Huber W, Gentleman R, Falcon S, Gentleman R, Carey VJ. Unsupervised Machine Learning. Bioconductor Case Studies. 2008;:137–57.

18. Csardi G, Nepusz T. The igraph software package for complex network research. *InterJournal Complex Systems* 2006;**Complex Sy**:1695.
